# Clustering Trend Changes of Lung Cancer Incidence in Europe via the Growth Mixture Model during 1990–2016

**DOI:** 10.1155/2021/8854446

**Published:** 2021-04-09

**Authors:** Mohammad Bahabin Boroujeni, Kamran Mehrabani, Hadi Raeisi Shahraki

**Affiliations:** ^1^Student Research Committee, Shahrekord University of Medical Sciences, Shahrekord, Iran; ^2^Department of Biostatistics, Faculty of Medicine, Shiraz University of Medical Sciences, Shiraz, Iran; ^3^Department of Epidemiology and Biostatistics, Faculty of Health, Shahrekord University of Medical Sciences, Shahrekord, Iran; ^4^Modeling in Health Research Center, Shahrekord University of Medical Sciences, Shahrekord, Iran

## Abstract

**Background:**

Lung cancer accounts for half of all deaths from cancer in Europe and has the highest incidence in Southern Europe. The current study aimed to cluster trend changes of lung cancer incidence in Europe via the growth mixture model.

**Methods:**

The dataset included incidence rates of female and male lung cancer per 100,000 for 42 European countries during 1990–2016 compiled from the Gapminder database. The growth mixture model was implemented to recognize different longitudinal patterns and estimate the linear trend of each pattern in Mplus 7.4 software.

**Results:**

The observed overall trend of incidence for female and male lung cancer was raising and falling, respectively, and Iceland was the only country with higher incidence of female versus male lung cancer in 2016. The growth mixture model suggests 3 main patterns for the trend of lung cancer incidence both for males and females. In male lung cancer, a sharp decreasing pattern was detected for 6 countries including Belarus, Estonia, Russia, Slovenia, Ukraine, and the United Kingdom; also, a moderately decreasing pattern was observed among the other countries. In female lung cancer, a moderate increasing trend was observed for 8 countries including the United Kingdom, Denmark, Hungary, Iceland, Ireland, Montenegro, Netherlands, and Norway; the other patterns were categorized into two clusters with slow increasing trends.

**Conclusion:**

Given the raising patterns in the incidence of lung cancer among European females, especially in the United Kingdom, Denmark, Hungary, Iceland, Ireland, Montenegro, Netherlands, and Norway, urgent effective measures are recommended to be taken.

## 1. Introduction

Lung cancer is now recognized as the most common male cancer in the Asian continent and has the highest incidence in South Europe and North America [1]. It also accounts for half of all deaths from cancer in Europe and is the second leading cause of cancer deaths among females [2, 3]. With 513,000 cases and 8.5% of all cancers, lung cancer accounts for fourteen most common cancer with a higher prevalence among males. In the Central and Eastern Europe, Hungary with 109, Macedonia with 102, Serbia with 99, and the Netherlands with 90 new cases per 100,000 annually had the highest incidence, and Finland with 45 and Sweden with 29 new cases per 100,000 annually had the lowest incidence rates of male lung cancer. Besides, Albania, Belarus, Bosnia and Herzegovina, Bulgaria, Greece, Hungary, Montenegro, the Netherlands, Romania, Serbia, and Ukraine, lung cancer have been the leading cause of new deaths among men [4].

Previous studies showed that the incidence of lung cancer in Hungary decreased by 1.3% from 1970 to 2005 in men and increased by 1.3% in women. In the same region (Eastern Europe), Romania has the highest increase with a growth rate of 1.5%. In northern Europe, the rate of lung cancer in men decreased by 2.5% between 1970 and 2007, and in England, it increased by 1% in women. South European women had one of the highest growth rates of lung cancer with a 3% increase in Spain and Slovenia. In the western European, during 1970–2007, France had a 0.9% reduction in lung cancer among men and a 4.2% increase in cancer among women. Germany also had a 2.3% increase in the rate of female lung cancer, but the highest rate of growth was in the Netherlands, which had a 4.6% increase in cancer rates among women [5]. Italy showed a 1.8% reduction in lung cancer among men and a 1.2% increase in women [5]; in Montenegro, an increase of 3.9% between the years 1990 and 2004 and a decrease of 1.95% between 2015 and 2015 was detected [6].

Growth mixture models (GMM) are known as capable statistical methods to identify and cluster different longitudinal patterns which have been more attentive recently. In GMM, diverse subgroups of trajectories can be modeled easily as a hidden variable which should be estimated [7]. To the best of our knowledge, there was no related previous study about modeling growth of lung cancer incidence through Europe during the last decades. Therefore, the current study aimed to cluster the trend changes of lung cancer incidence in Europe via the growth mixture model.

## 2. Materials and Methods

In this study, our dataset included the incidence rates of female and male lung cancer per 100,000 for 42 European countries during 1990–2016 compiled from the Gapminder database that is freely available at http://www.gapminder.org/data. The growth mixture model was implemented to recognize different longitudinal patterns and estimate linear trend of each pattern. In the statistical modeling, the main outcome was the annual incidence of lung cancer for males or females at each country.

Using growth mixture models to cluster trajectories in cancer epidemiology have been more attentive in the last decades. In the study of Borumandnia et al., GMM was used to find the main patterns of mortality among Iranian men and women due to various cancers [8]. Salari et al. suggest GMM to cluster trends in the liver cancer mortality in Asia from 1990 to 2015, and Zayeri et al. performed GMM to determine the new clusters of colon and rectum cancer mortality in Asia and North Africa region [9, 10]. In the current study, the growth mixture model with different number of clusters (patterns) was fitted, and the most appropriate model was estimated based on the *P* value of the likelihood ratio test (LRT) in Mplus 7.4 software.

## 3. Results

**Figure 1:**
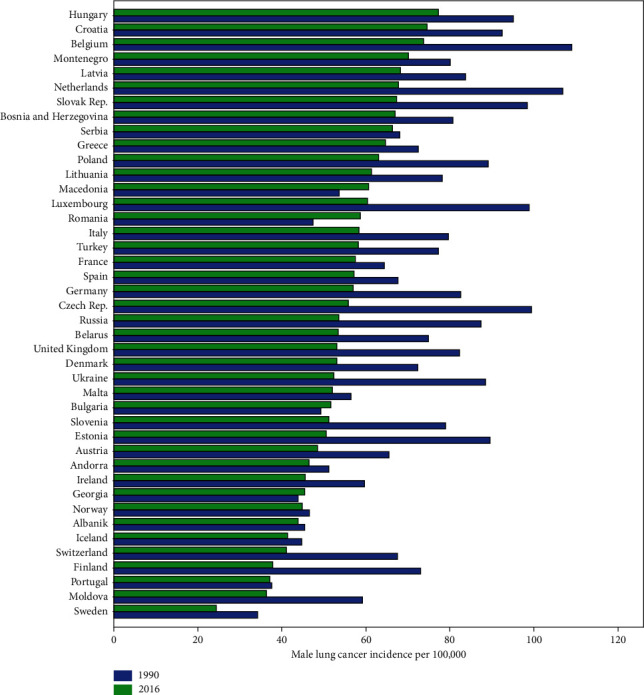
Bar chart of male lung cancer incidence among European countries.

**Figure 2:**
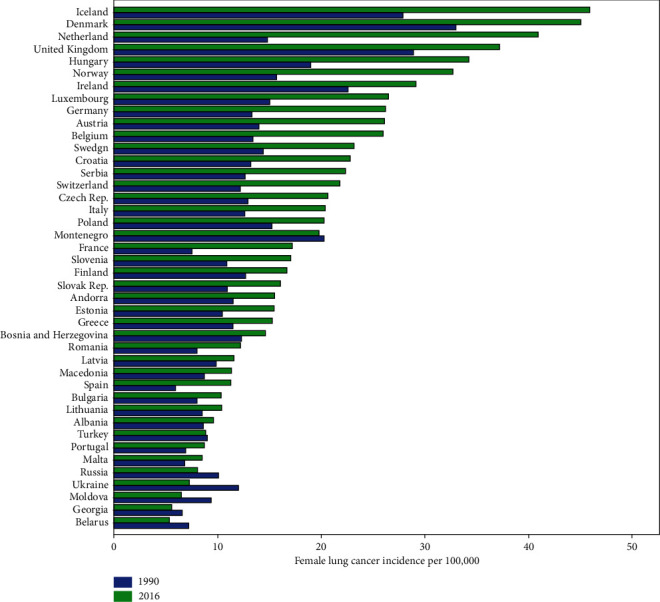
Bar chart of female lung cancer incidence among European countries.

**Figure 3:**
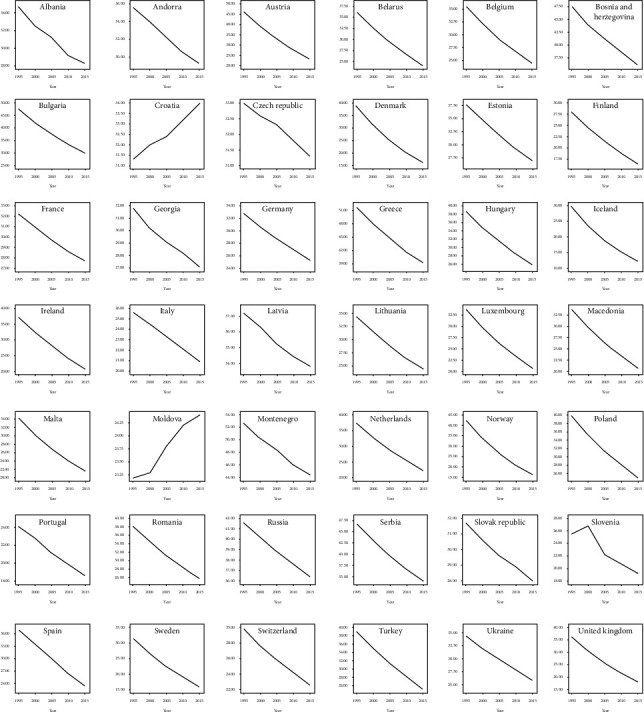
The trend changes of tobacco smoking prevalence in European countries.

Longitudinal trajectories of lung cancer in 42 European countries for both males and females were monitored during 1990–2016. Romania and Netherlands had experienced the highest increase in the incidence of male and female lung cancer, respectively. Also, Finland and Ukraine showed the highest decrease in the incidence of male and female lung cancer, respectively, during the period of the study. Iceland was the only country with higher incidence of female versus male lung cancer in 2016 (Figures 1 and 2). Also, the trend changes of tobacco smoking prevalence for all of the 42 European countries are shown in Figure 3. The observed trajectories were upward for Croatia and Moldova but downward for the other countries.

Although the observed overall trend of the incidence for female and male lung cancer was raising and falling, respectively, we implemented the growth mixture model to identify different trajectories. The growth mixture model with 3 clusters was chosen as the most appropriate for both male and female lung cancer modeling based on the LRT test (Tables 1 and 2). Also, related information about each recognized pattern including the number of countries, incidence rate in 1990 (intercept), and annual trend change (slope) of estimated linear trend is summarized in Table 3.

**Figure 4:**
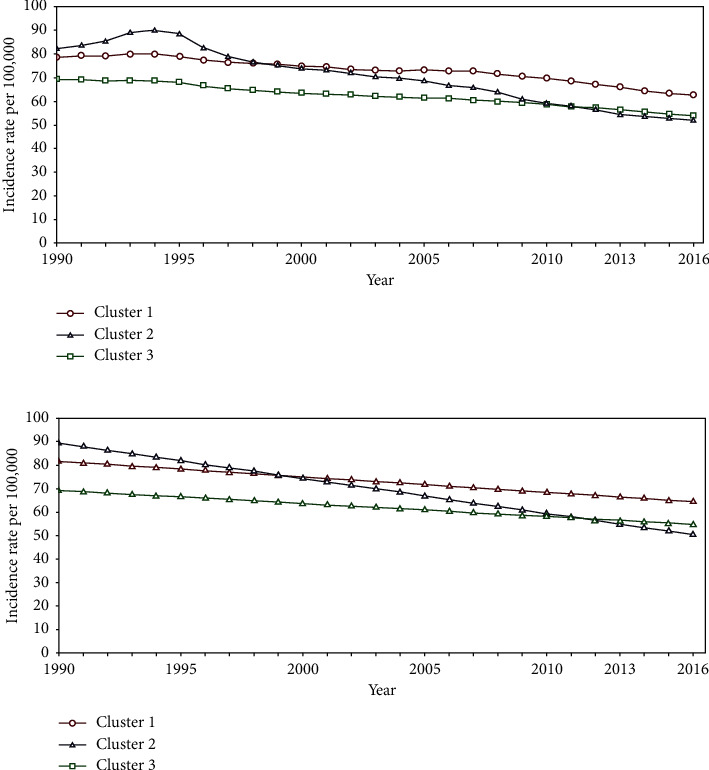
The overall mean (top) and estimated linear trend (bottom) for male lung cancer. Cluster 1, Andorra, Croatia, Hungary, Lithuania, Norway, Slovak Republic, Spain, and Turkey. Cluster 2, Belarus, Estonia, Russia, Slovenia, Ukraine, and the United Kingdom. Cluster 3, other European countries.

**Figure 5:**
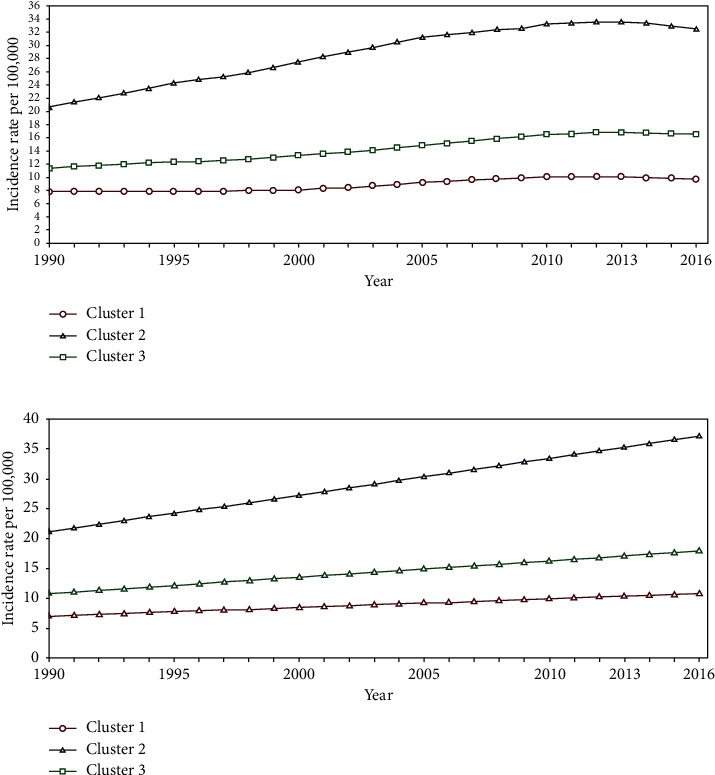
The overall mean (top) and estimated linear trend (bottom) for female lung cancer. Cluster 1, Bulgaria, Georgia, Latvia, Macedonia, Malta, Portugal, and Romania. Cluster 2, the United Kingdom, Denmark, Hungary, Iceland, Ireland, Montenegro, Netherlands, and Norway. Cluster 3, other European countries.

For male lung cancer, 8 countries including Andorra, Croatia, Hungary, Lithuania, Norway, Slovak Republic, Spain, and Turkey belonged to cluster 1. Annual slope of −0.66 per 100,000 showed a moderate decrease in these countries. In the second cluster, slope of −1.5 per 100,000 indicated a sharp falling pattern for Belarus, Estonia, Russia, Slovenia, Ukraine, and the United Kingdom countries, and another 28 countries belonged to cluster 3 which had experienced an annual change of −0.56 per 100,000 at male lung cancer (Figure 4). Modeling trend changes of female lung cancer assigned Bulgaria, Georgia, Latvia, Macedonia, Malta, Portugal, and Romania countries to a cluster with almost a constant or very slow increasing pattern of incidence over the study period. The second cluster including the United Kingdom, Denmark, Hungary, Iceland, Ireland, Montenegro, Netherlands, and Norway showed a moderate increasing trend, and another 27 countries belonged to cluster 3 with slow raising trends (Figure 5).

## 4. Discussion

Time trends of lung cancer showed falling and raising patterns for males and females, respectively, among most of the European countries. Our results suggest 3 main patterns for the trend of lung cancer incidence both for males and females. In male lung cancer, a sharp decreasing pattern was detected for 6 countries including Belarus, Estonia, Russia, Slovenia, Ukraine, and the United Kingdom and a moderately decreasing pattern was observed among the other countries. In female lung cancer, a moderately increasing trend was observed for 8 countries including the United Kingdom, Denmark, Hungary, Iceland, Ireland, Montenegro, Netherlands, and Norway; the other patterns were categorized into two clusters with slow increasing trends.

As it was mentioned by Aareleid, the peak of male lung cancer incidence in Estonia was observed at 1991 and decreased thereafter. As a justification, the authors have mentioned that growing public awareness and stricter tobacco control have stimulated overall favorable changes in men, but not yet in women. They also report an overall increasing trend of lung cancer incidence among women which may be due to a substantial increase of tobacco smoking, particularly among women, after the Second World War [11]. In line with our results, the lung cancer incidence reports in Russia indicate an increasing incidence rate among women and a decreasing pattern among men. The observed pattern for women is unfavorable because it shows a rising burden of smoking attributable disease in females [12]. Peto et al. announced that in the UK, women and older men who were still current smokers in 1990 were more likely than those in 1950 to have been persistent cigarette smokers throughout adult life and so had higher lung cancer rates than the current smokers in 1950 [13].

A previous study about the incidence of lung cancer in Hungary during 1970–2005 showed that the yearly increase in the incidence of LC decreased, as the results of our study, characterized by a public perception that smoking is a health hazard, allowing the introduction of comprehensive tobacco control legislation [14]. However, related studies showed that in countries such as Hungary and Poland, the average number of cigarettes smoked per person per day is higher than any member country of the EU. High lung cancer rates around the year 2000 may also be associated with higher rates of cigarette smoking in the 1980s (and before) in some EUCSs, particularly in the Baltic countries [15].

In Spain, the trend of lung cancer increased among women, as we showed. Diverging trends in the prevalence of smoking could explain the increase in the rate of lung cancer-related mortality among Spanish women since the early 1990s [16]. In the Portugal, as we found, the rate of lung cancer decreased in men and increased in women between 1955 and 2005. These results place Portugal at the end of the third stage of the smoking epidemic [17]. In Belgium, the study of Van Hemelrijck et al. showed that lung cancer was decreased and increased for men and women, respectively, during 1954–1997. Both prevalence and consumption declined for Belgian males. In contrast, female cigarette consumption increased over time, while prevalence remained rather stable (about 20%) during the early 1990s. The same results were also reported in Netherlands. Female lung cancer in all age groups showed a significant annual increase in lung cancer during 1950–2000, which was consistent with our results [18].

Formal statistics in Switzerland approved that decrease in male lung cancer mortality was 20% over the last decade (from 42.9 to 34.3/100 000). In contrast, lung cancer mortality in women has steadily increased by 38% between 1981 and 1991 and by 47% between 1991 and 2001 to reach 10.7/100 000 at all ages and 18.3 at age 35–64, due to increased prevalence of smoking in subsequent generations of Swiss women [19]. Szczuka and Roszkowski-Śliż found that lung cancer incidence and mortality rate in men has been on the stable level, even with the tendency to decline in the last decade in Poland. Incidence and mortality rates among women have continued to increase although the trend of increase has slowed in the last years, as we showed. Despite some improvement of epidemiological situation in lung cancer in Poland, it still remains the most common malignancy in men and is on the third position in the most frequent cancers in woman [20]. Increasing trend of female lung cancer in the period of 1988–1992 was reported in Bulgaria by Hristova et al. [21]. Also, the study of Eilstein et al. in France found that during 1978–2002, female lung cancer mortality rate increased by 3.3% annually. For men, a slow increase was observed from 1988 to 1992, followed by a declining trend. Their results highlight the relevance of pursuing public health measures in order to cope more actively with tobacco smoking in the prevention strategy against lung cancer, specifically among women [22].

Study of Levi et al. in Germany showed that lung cancer rates in young women rose from 0.8 to 1.0/100,000 in the early 1970s to 1.7–1.9 in the mid-1990s and levelled off during the last decade [23]. Moreover, based on the study of Yilmaz, the increase in the cancer incidence is much faster in men than women in Turkey, further widening the gap between the incidence among men and women. An increase in tobacco consumption was paralleled some 20–30 years later by an increase in the incidence of lung cancer; similarly, a decrease in consumption is followed by a decrease in incidence [24]. A meta-analysis study in Turkey showed that the rate of LC in females increased significantly in the last decade which may be due to increasing prevalence of smoking in women. The prevalence of smoking in Turkey has increased during the past 3 decades. Cigarette consumption increased by 10% from 1970 to 1985; however, this consumption went up to 44% in 1988 with a smoking prevalence of 63% for males and 24% for females. Additionally, it has been suggested that female smokers are more susceptible to lung cancer than male smokers. When compared to the period 1986–1995, the ratio of males decreased from 93.3 to 91.8. However, the mean age increased from 58.5 to 59.8 years in the period of 1996–2005 [25].

Divergent trends among countries highlight the other possible risk factors such as racial disparities in nonadjacent counties [26], exposure to particulate matter air pollution [27], and exposure to second-hand smoke [28]. Moreover, the previous studies confirm a significant inverse association was found between low education and lung cancer risk in men in Central Europe [28, 29]. Finally, the downward trends of male lung cancer in European countries may be due to substantial decrease in dangers occupational exposures such as arsenic and asbestos during the last decades [28, 30, 31].

To the best of our knowledge, a few numbers of studies were devoted to assess the trend changes of lung cancer incidence in Europe. This study tries to apply a highly advanced model along with the most recent epidemiological data about lung cancer but lack of statistical modeling studies in this field and accurate descriptive statistics about the incidence rates in a few number of less developed countries caused limitations in comparing obtained findings in this study.

## 5. Conclusion

Given the raising patterns in the incidence of lung cancer among European females, especially in the United Kingdom, Denmark, Hungary, Iceland, Ireland, Montenegro, Netherlands, and Norway, urgent effective strategies seem to be necessary.

## Figures and Tables

**Table 1:** Fit indices to estimate the best model for clustering European male lung cancer.

Fit indices	Number of cluster					
	1	2	3	4	5	6
AIC	5752	5751	5745	5752	5760	5757
BIC	5808	5815	5818	5834	5850	5856
SSBIC	5708	5699	5687	5687	5687	5678
LRT *P* value	—	0.24	<0.001	0.55	0.53	0.36

**Table 2:** Fit indices to estimate the best model for clustering European female lung cancer.

Fit indices	Number of cluster					
	1	2	3	4	5	6
AIC	3059	3034	3026	3029	3050	3053
BIC	3115	3098	3099	3111	3140	3152
SSBIC	3014	2982	2968	2963	2978	2973
LRT *P* value	—	0.23	0.09	0.69	0.91	0.50

**Table 3:** Intercept and slope of the estimated linear trend for each cluster of the growth mixture model.

Gender	Cluster	Number of countries	Intercept		Slope	
			Estimate	SE	Estimate	SE
Male	1	8	81.66	11.95	−0.66	0.21
	2	6	89.45	3.85	−1.50	0.13
	3	28	69.31	4.06	−0.56	0.12

Female	1	7	7.02	0.47	0.15	0.02
	2	8	21.10	3.23	0.62	0.13
	3	27	10.79	0.59	0.27	0.06

## Data Availability

The data used to support the findings of this study are available at Gapminder website (https://www.gapminder.org/data/).
